# Glioblastoma gene network reconstruction and ontology analysis by online bioinformatics tools

**DOI:** 10.1515/jib-2021-0031

**Published:** 2021-11-16

**Authors:** Natalya V. Gubanova, Nina G. Orlova, Arthur I. Dergilev, Nina Y. Oparina, Yuriy L. Orlov

**Affiliations:** Institute of Cytology and Genetics, Siberian Branch of the Russian Academy of Sciences, 630090 Novosibirsk, Russia; Financial University under the Government of the Russian Federation, 119991 Moscow, Russia; Moscow State Technical University of Civil Aviation, 125993 Moscow, Russia; Novosibirsk State University, 630090 Novosibirsk, Russia; University of Gothenburg, 405 30 Gothenburg, Sweden; The Digital Health Institute, I.M.Sechenov First Moscow State Medical University of the Russian Ministry of Health, 119991 Moscow, Russia

**Keywords:** drug search, gene networks, gene ontology, glioblastoma, medical genomics

## Abstract

Glioblastoma is the most aggressive type of brain tumors resistant to a number of antitumor drugs. The problem of therapy and drug treatment course is complicated by extremely high heterogeneity in the benign cell populations, the random arrangement of tumor cells, and polymorphism of their nuclei. The pathogenesis of gliomas needs to be studied using modern cellular technologies, genome- and transcriptome-wide technologies of high-throughput sequencing, analysis of gene expression on microarrays, and methods of modern bioinformatics to find new therapy targets. Functional annotation of genes related to the disease could be retrieved based on genetic databases and cross-validated by integrating complementary experimental data. Gene network reconstruction for a set of genes (proteins) proved to be effective approach to study mechanisms underlying disease progression. We used online bioinformatics tools for annotation of gene list for glioma, reconstruction of gene network and comparative analysis of gene ontology categories. The available tools and the databases for glioblastoma gene analysis are discussed together with the recent progress in this field.

## 1 Introduction

Glioblastomas, along with astrocytomas, are the most common primary tumors of the central nervous system and make up approximately 60–70% of all pure brain tumors, challenging search for new therapy methods. Primary glioblastoma in 60% of cases occurs in people over 50 years old, while secondary glioblastoma is more characteristic for people under 45 years old [1, 2]. Glioblastoma is characterized by rapid infiltrative growth, the presence of foci of necrosis, a change in blood vessels. The lack of clear boundaries and the ability to relapse are a particular problem for surgical removal of the tumor, and for immunotherapy [3]. Primary central nervous system tumors are relatively rare and account for approximately 2% of all oncological diseases. However, tumors of the brain and spinal cord are the second most common form of malignancy in children after leukemia [4]. Glioblastoma has higher burden in survival of the patients with malignant brain tumors [5]. Despite the development in early detection, and treatment, 5-year glioblastoma survival only increased from 4 to 7% in past 4 decades [5]. Gliomas are the most common heterogeneous group of malignant tumors of the brain among the adult population, which differ in morphological characteristics, clinical course, and response to radio- and chemotherapy. The incidence of gliomas is 3–5: 100,000 of the adult population. In the treatment of glioblastoma with drugs, an obstacle is its resistance to a number of antitumor drugs initially, as well as the presence of a blood-brain barrier that negates the effectiveness of most chemotherapeutic agents and targeted drugs [3, 6].

For an object such as gliomas, it is necessary to conduct new studies based on modern cellular technologies, genome-wide technologies for high-throughput sequencing, and the integration of available information from international databases and genomic projects [7, 8].

The practical task was to construct (collect) a list of genes associated with the development of glioblastoma, analyze the categories of gene ontologies for such a list, and reconstruct the gene network. For key genes of the disease, obtained by analyzing the structure of the gene network, options for searching for drugs (substances that interact with this protein) will be considered.

There are open international databases on gene expression including glioblastoma (microarrays and sequencing data of GEO NCBI, http://www.ncbi.nlm.nih.gov/geoprofiles/), expression in various types of tumor cells (The Cancer Gene Atlas, cancergenome.nih.gov), gene expression for brain compartments (Allen Brain Atlas), protein interactions databases such as HPRD (http://hprd.org/), KEGG biochemical reactions (http://www.genome.jp/kegg/), Interactome (http://interactome.org/), sequenced tumor genomes, including gliomas and glioblastomas (https://cghub.ucsc.edu/). The Allen Institute has developed the Ivy Glioblastoma Atlas Project database (http://glioblastoma.alleninstitute.org/) according to patients with glioma. We demonstrate that these publicly available online bioinformatics tools can give helpful information for annotation of gene list for glioblastoma, reconstruction of gene network and comparative analysis of the related diseases. This approach for gene network analysis of list of gene names using online bioinformatics tools presents application of bioinformatics methods to annotation of complex human diseases.

## 2 Methods

Obtaining a list of genes associated with a hereditary predisposition to glioblastoma. The Internet resource OMIM (Online Mendelian Inheritance in Man) (https://omim.org/, access date 10.10.2021) was used to analyze the genes of Mendelian inheritance in humans [9]. Search for the keyword ‘glioblastoma’ gave 264 genes. As a result, 264 gene names (official gene symbols) were obtained (ADAM10, ADAM17, ADAM9, ADGRB1, etc., see the Supplement).

Next, we used DAVID (Database for Annotation, Visualization and Integrated Discovery) tool (https://david.ncifcrf.gov/summary.jsp) [10] and the PANTHER (Protein ANalysis THrough Evolutionary Relationships) (http://pantherdb.org/) resource [11] for gene ontology analysis. Then we applied gene network reconstruction tools described in the next sections.

**Figure 1:**
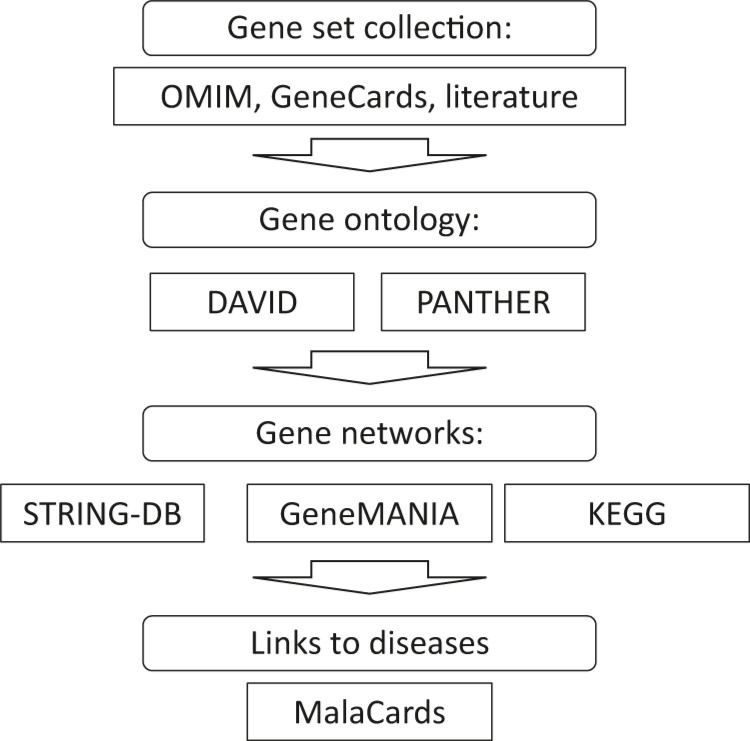
Workflow for information data processing for glioblastoma genes.

The workflow of the data processing is presented in Figure 1.

Figure 1 shows main steps of data processing. Data collection starts from the OMIM database (https://omim.org/) and GeneCards (https://www.genecards.org/) resources. At next step gene ontology analysis tools PANTHER and DAVID are used as well as g:Profiler (http://biit.cs.ut.ee/gprofiler/gost) online tool. Then STRING-DB (https://string-db.org) and GeneMANIA (https://genemania.org/) online applications help in the network reconstruction and visualization. Finally MalaCards (https://www.malacards.org/) and ToppGene (https://toppgene.cchmc.org/) allow find disease-disease interactions and prioritize genes in the list.

## 3 Results

We have analyzed gene ontology categories for the same gene list obtained by different online bioinformatics tools. DAVID [10] and the PANTHER resource [11] were used to estimate the categories of gene ontologies.

### 3.1 Comparison of gene ontology categories

A list of 264 human genes was loaded through the DAVID interface to search for relevant categories of gene ontologies for this group of genes. 255 identifiers were used next (the Functional Annotation Chart option).

The table was limited to values of normalized *p*-value <0.00001 (or, in power notation, <1.0 × 10^−5^) (Bonferroni correction). We deleted categories (table rows) for groups of less than eight genes, and Fold enrichment (observed number of genes/expected) less than 2.

As can be seen from Table 1, the most significant GO categories for glioblastoma genes are protein binding, positive regulation of protein phosphorylation, membrane location (according to cellular compartments – GOTERM_CC classification), and kinase activity. In general, according to the categories from the table, membrane proteins and kinases play an important role, apparently associated with signal transduction into the cell. UP_KEYWORD (terms from UniProt (https://www.uniprot.org/) shows associations with tumors and kinase activity.

**Table 1:** Categories of gene ontologies for glioblastoma genes according to DAVID

Category set	Term	# genes	Fold	Bonferroni
UP_KEYWORDS	Tumor suppressor	24	12	1.3 × 10^−15^
UP_KEYWORDS	Phosphoprotein	154	1.6	3.0 × 10^−13^
UP_KEYWORDS	Disease mutation	71	2.5	1.2 × 10^−10^
GOTERM_MF_DIRECT	Protein binding	164	1.5	4.2 × 10^−10^
GOTERM_BP_DIRECT	Negative regulation of cell proliferation	26	5	1.8 × 10^−7^
INTERPRO	Protein kinase-like domain	28	4.3	2.1 × 10^−7^
GOTERM_BP_DIRECT	Positive regulation of protein phosphorylation	16	9.6	2.5 × 10^−7^
GOTERM_MF_DIRECT	Protein kinase binding	24	5	2.6 × 10^−7^
UP_KEYWORDS	Kinase	31	3.7	3.1 × 10^−7^
UP_KEYWORDS	Proto-oncogene	18	6.7	4.4 × 10^−7^
UP_KEYWORDS	Cell cycle	27	3.7	6.6 × 10^−6^
UP_KEYWORDS	Tyrosine-protein kinase	12	9.5	1.5 × 10^−5^
INTERPRO	Protein kinase, catalytic domain	24	4	1.8 × 10^−5^
UP_SEQ_FEATURE	Binding site:ATP	25	3.9	3.2 × 10^−5^
GOTERM_MF_DIRECT	Kinase activity	17	5.5	4.0 × 10^−5^
UP_SEQ_FEATURE	Active site:Proton acceptor	27	3.5	6.8 × 10^−5^
GOTERM_BP_DIRECT	Protein phosphorylation	24	4	7.7 × 10^−5^
GOTERM_MF_DIRECT	Protein kinase activity	20	4.3	8.7 × 10^−5^
GOTERM_CC_DIRECT	Membrane	56	2	9.6 × 10^−5^

Gene ontologies were analyzed for the same list of genes using the PANTHER resource (Protein ANalysis THrough Evolutionary Relationships) [11]. Table 2 of ontologies for categories of biological processes was built using PANTHER.

**Table 2:** Categories of gene ontologies for glioblastoma genes according to PANTHER (biological process).

GO categories (biological process complete)	# genes	Fold enrich.	*p* value
Negative regulation of biological process	146	2.36	2.21 × 10^−25^
Negative regulation of cellular process	137	2.40	2.20 × 10^−23^
Anatomical structure development	136	2.28	5.27 × 10^−21^
System development	123	2.47	7.48 × 10^−21^
Regulation of cell population proliferation	75	3.81	3.21 × 10^−20^
Developmental process	142	2.14	1.42 × 10^−19^
Regulation of signaling	102	2.62	1.39 × 10^−17^
Regulation of cell differentiation	67	3.75	3.90 × 10^−17^
Regulation of cell death	69	3.63	4.97 × 10^−17^
Regulation of apoptotic process	64	3.76	3.44 × 10^−16^
Cellular developmental process	103	2.49	4.11 × 10^−16^
Regulation of programmed cell death	64	3.68	9.59 × 10^−16^
Regulation of signal transduction	91	2.66	3.02 × 10^−15^
Regulation of cellular component movement	53	4.33	3.48 × 10^−15^
Nervous system development	78	2.98	5.86 × 10^−15^
Regulation of epithelial cell proliferation	32	7.93	1.10 × 10^−14^
Positive regulation of phosphorus metabolic process	47	4.44	1.96 × 10^−13^
Immune system development	40	5.04	1.21 × 10^−12^
Regulation of nervous system development	33	6.31	1.69 × 10^−12^
Generation of neurons	53	3.59	7.61 × 10^−12^

In total, 238 identifiers were recognized from the same list of genes, the others were not recognized or could not be unambiguously mapped. In total, 20,595 genes were used in the PANTHER reference genome (15 October 2021). We limited the output to *p*-values up to 10^−12^ (after Bonferroni correction) and Fold enrichment greater than two to represent the most informative results. Table 2 shows that the most significant categories for glioblastoma genes are negative regulation of biological processes, developmental processes, generation of neurons, and cell proliferation.

The categories of processes related to the development of the nervous system are also presented (this is expected, since glioblastoma is a brain disease).

Further, the categories of gene ontologies for molecular functions and cellular compartments were calculated using PANTHER (Table 3). The threshold for the significance of the categories was taken already at the 10^−3^ level (after the Bonferroni correction).

**Table 3:** Categories of gene ontologies for glioblastoma genes according to PANTHER (molecular functions and cellular compartments).

	# genes	Fold enrich.	*p* value
**GO categories – molecular function complete**			
Protein binding	225	1.31	7.98 × 10^−13^
Enzyme binding	68	2.76	2.84 × 10^−8^
Kinase binding	36	3.94	1.54 × 10^−8^
Phosphotransferase activity alcohol group as acceptor	31	3.76	1.57 × 10^−6^
Protein tyrosine kinase activity	14	8.20	1.61 × 10^−5^
Transferase activity transferring phosphorus-containing groups	34	3.09	2.78 × 10^−5^
Beta-catenin binding	9	8.66	5.97 × 10^−3^
Growth factor receptor binding	11	6.49	6.20 × 10^−3^
**GO categories – cellular component complete**			
Organelle lumen	115	1.74	6.06 × 10^−8^
Cytosol	111	1.72	3.16 × 10^−7^
Cell junction	59	2.35	8.77 × 10^−7^
Cell projection	63	2.23	1.75 × 10^−6^

The results for cellular components show that the most significant categories for glioblastoma genes are organelles, cell junction. It may be associated with signal transmission between cells and refers to neurons.

Thus, both DAVID and PANTHER for glioblastoma genes support the categories of gene ontologies of protein binding, membranes, cell adhesion, and intercellular contacts. Note that direct comparison of DAVID and PANTHER tools proved be limited due to different database version and genome annotation versions [12]. Other available resources for calculating gene ontologies – GeneOntology (http://geneontology.org/) and g:Profiler GOST (http://biit.cs.ut.ee/gprofiler/), which are not presented in this study, can also be used.

### 3.2 Reconstruction of gene networks for glioblastoma genes

**Figure 2:**
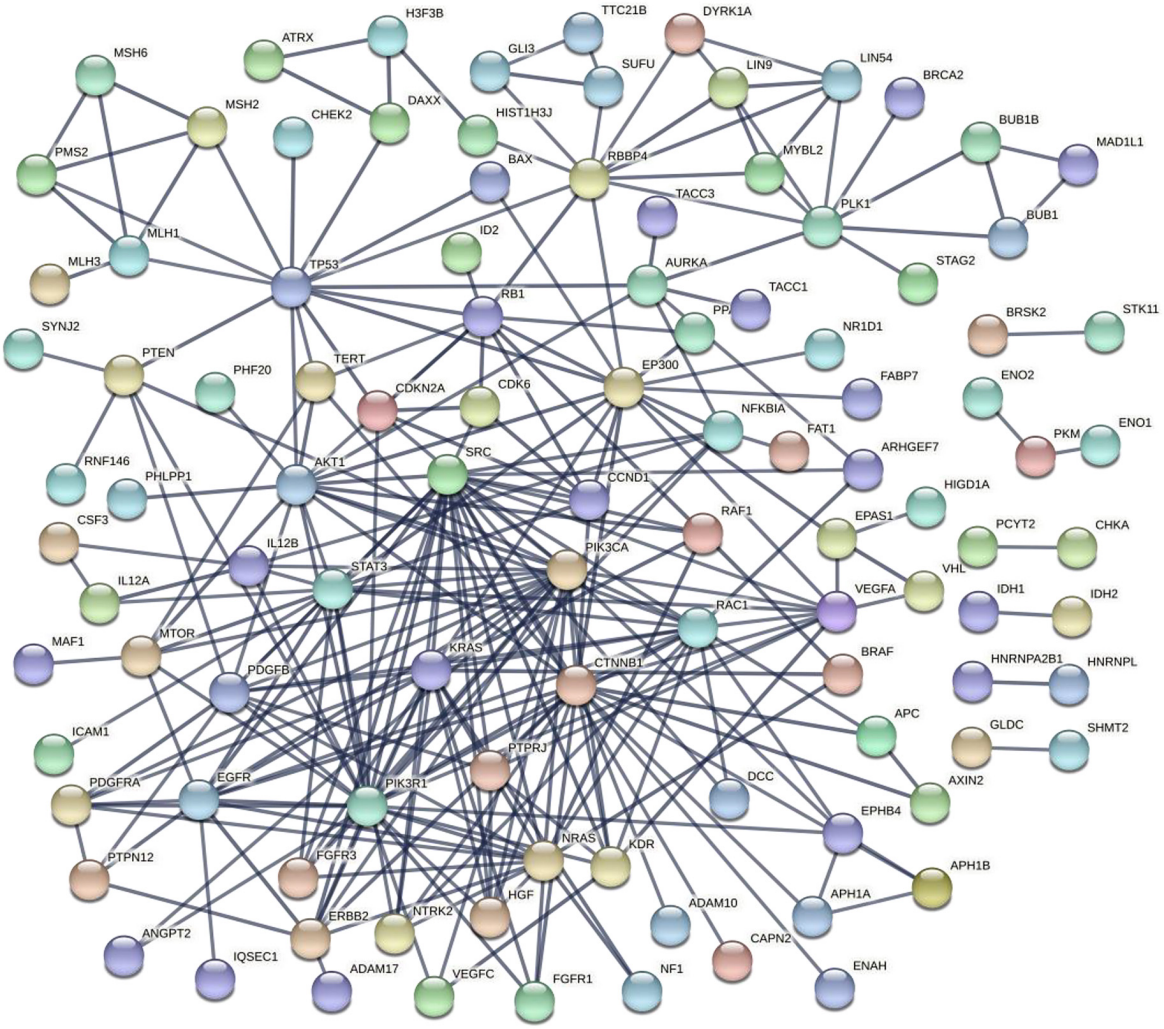
Gene network for glioblastoma genes reconstructed by STRING-DB.

To reconstruct the gene network of interactions between glioblastoma genes, the resources of GeneMANIA (https://genemania.org/) and STRING-DB (https://string-db.org/) were used. The following Figure 2 shows the glioblastoma gene network reconstructed with GeneMANIA and STRING-DB.

In the centre of the network there are the genes (proteins) of glioblastoma, which have a large number of connections with other elements – PTRN, TP53, KRAS.

Consider the results of network reconstruction using STRING-DB (https://string-db.org/) for the same list of glioblastoma genes (Figure 2). It can be seen that the network is quite sparse, some objects (proteins) do not contact others. A central, strongly linked cluster of genes is highlighted.

In total, 243 genes from the list were recognized by STRING-DB. Statistics on STING-DB show that the network has a reasonably large number of connections (with a significance <1.0 × 10^−16^), the average degree of connectivity of the network node (protein) is 13.6, the clustering coefficient is 0.5. The drawing from the STING-DB was iteratively rebuilt with the removal of unrelated genes and the replacement of several colored connections between the nodes of the network – with one line (confidence), taking into account only experimentally proven interactions. Than we increase parameter ‘confidence level’ to keep only verified links in the network.

The figure shows several hubs of the network – the largest includes genes RBBP4, AKT1, MTOR, KRAS, STAT3, PTEN, several smaller, but also connected in a common network of clusters, the largest number of connections in the TP53 gene (a well-known oncogene) can be seen.

In general, the analysis of the structure of the gene network for glioblastoma genes shows the existence of a dense, connected, sufficiently large cluster of genes (network nodes) including known oncogenes, such as TP53.

Thus, standard online bioinformatics tools allow to model main parameters of gene network related to given disease. This approach is quite useful in distant education for medical students due to open access tools, fast visualization, interactive user interface not demanding programming. Integration of the results obtained by online bioinformatics tools and genetics data leads to novel knowledge in the study of complex oncological diseases.

## 4 Related works

Let’s consider functional annotation of the glioblastoma genes using different data sources and their combinations.

According to GeneCards (genecards.org), the following 20 genes are the most significant.

Table 4 shows large fraction of non-protein coding genes in the list. The score in the table column show relevance of the gene to the search query to the literature database (https://www.genecards.org/Guide/Search#relevance). Lager the GeneCards score, higher relevance to the disease is. GeneCards Inferred Functionality Scores (GIFtS) parameter shows gene functionality annotated in the databases (https://www.genecards.org/Guide/GeneCard#GIFtS) [13]. Note that the hub genes in the network have high functionality, while RNA less annotated and included in standard gene network (in STRING-DB).

**Table 4:** Gene list for glioblastoma by GeneCards.

	Symbol	Description	Category	GIFtS	Relevance score
1	TP53	Tumor protein P53	Protein coding	52	52.29
2	EGFR	Epidermal growth factor receptor	Protein coding	52	40.05
3	IDH1	Isocitrate dehydrogenase (NADP(+)) 1	Protein coding	51	38.95
4	MSH2	MutS homolog 2	Protein coding	48	33.76
5	MGMT	O-6-Methylguanine-DNA methyltransferase	Protein coding	48	31.36
6	ERBB2	Erb-B2 receptor tyrosine kinase 2	Protein coding	52	29.57
7	MIR21	MicroRNA 21	RNA gene	24	28.37
8	BRCA2	BRCA2 DNA repair associated	Protein coding	48	27.59
9	PTEN	Phosphatase and tensin homolog	Protein coding	51	26.68
10	PIK3CA	Phosphatidylinositol-4,5-bisphosphate 3-kinase	Protein coding	52	25.74
		catalytic subunit alpha			
11	MIR221	MicroRNA 221	RNA gene	21	24.00
12	MIR222	MicroRNA 222	RNA gene	21	23.89
13	FGFR1	Fibroblast growth factor receptor 1	Protein coding	53	22.81
14	MIR34A	MicroRNA 34a	RNA gene	22	22.63
15	MIR296	MicroRNA 296	RNA gene	17	22.51
16	H3-3A	H3.3 histone A	Protein coding	34	21.76
17	MIR137	MicroRNA 137	RNA gene	19	44.398
18	IDH2	Isocitrate dehydrogenase (NADP(+)) 2	Protein coding	51	20.63
19	PPARG	Peroxisome proliferator activated receptor gamma	Protein coding	51	20.37
20	MIR326	MicroRNA 326	RNA gene	21	20.14

Prioritization of genes was performed using the resource ToppGene: Candidate gene prioritization (https://toppgene.cchmc.org) [14].

The genes presented were found to be associated with the following diseases (Table 5).

**Table 5:** Diseases found by prioritization of shared genes to glioblastoma.

No.	Description	Category	Score
1	Glioma	1.882 × 10^−25^	9.333 × 10^−22^
2	Pilocytic astrocytoma	3.147 × 10^−25^	1.561 × 10^−21^
3–4	Adult pilocytic astrocytoma/childhood pilocytic astrocytoma	1.829 × 10^−22^	9.069 × 10^−19^
5	Malignant glioma	3.741 × 10^−21^	1.855 × 10^−17^
6	Mixed gliomas	3.741 × 10^−21^	1.855 × 10^−17^
7	Neurofibromatosis 1	6.750 × 10^−21^	3.348 × 10^−17^
8	Malignant neoplasm of soft tissue	2.560 × 10^−20^	1.270 × 10^−16^
9	Ganglioglioma	4.231 × 10^−20^	2.098 × 10^−16^
10–11	Childhood oligodendroglioma/adult oligodendroglioma	4.616 × 10^−20^	2.290 × 10^−16^
12	Sarcoma	2.272 × 10^−19^	1.127 × 10^−15^

One can see in the table relevant diseases categories related to brain, glioma types and soft tissues cancers.

The approaches of gene ontology analysis and gene network reconstruction using similar bioinformatics tools to search for glioma hub genes are published in series of papers. In recent work by [15] hub genes in the tumor microenvironment were identified using TCGA data. The hub genes for glioblastoma were identified by the Cytoscape tool (https://cytoscape.org/), and pathway enrichment analysis of the genes was performed using Database for Annotation, Visualization and Integrated Discovery (DAVID). Functional enrichment analysis identified set of upregulated and downregulated cross genes, which were mainly linked to immune response, inflammatory response, cell membrane, and receptor activity. Li and co-authors [16] used weighted gene co-expression network analysis to define hub genes.

Gene Set Enrichment Analysis (GSEA) identified further hub genes-related pathways. The Cancer Genome Atlas (TCGA) database was used to identify differentially expressed genes [17]. Then Gene Ontology and Kyoto Encyclopedia of Genes and Genomes (KEGG) analyses were used to determine the related functions and pathways of these genes.

Such analysis can lead to the identification of potential target genes and related drugs. Yang and Yang [18] recently analyzed glioma genes by GSEA, and the significantly enriched KEGG pathways involved in synapse signaling and oxytocin signaling pathways. The core molecules of GBM and the DrugBank database were assessed to identify 10 drugs included tetrachlorodecaoxide related to cancer and neuropsychiatric diseases [18].

## 5 Conclusions

After years of intensive research glioblastoma remains a dangerous disease with a low survival rate. The existing methods of therapy (chemotherapy, radio- and immunotherapy) can prolong the patient’s life without remission and glioblastoma data integration and analysis can lead to promising therapeutic targets [19].

The study of the structure of the gene network shows a high connectivity of genes and their products. Glioma progression is strongly connected with different types of epigenetic phenomena, such as histone modifications, DNA methylation, chromatin remodeling, and aberrant microRNA [18]. Amid the epigenetic therapies, histone deacetylase inhibitors (HDACIs) and DNA methyltransferase inhibitors have been used for treating tumors [20].

Long non-coding RNAs, circular RNAs, and transcribed pseudogenes act as ceRNA (competing endogenous RNA) to regulate the expression of related genes by sponging the shared microRNAs [21]. Circular (circ)RNAs serve important roles in the development and progression of glioma and may have potential as therapeutic targets too [22].

A set of glioma-related genes obtained from the Comparative Toxicogenomics Database were predicted to be regulated by 15 miRNAs via the miRwalk 2.0 database [22]. Zhu and colleagues [23] provided a framework of workflow for potential therapeutic drug discovery and predicted 10 potential drugs for glioblastoma therapy. Effectiveness of drugs has to be tested on the experimental models [24]. Chemotherapy for glioblastoma remains ineffective due to insufficient penetration of therapeutic agents into the tumor [25]. Recent works show limits on the isotope distribution in tissues in a glioma xenograft animal model [26].

An analysis of the literature (PubMed) showed continued growth of publications on the topic – there are about 54 thousand publications up to date. Integration of large biological databases and re-analysis, combination of different tools allows keeping current structure of the disease description and annotation, as we have discussed at the international conferences series on computational genomics [27, 28]. Network analysis of interacting genes shows existing gene clusters in the network, and potential new targets for therapy [29]. Overall, such application of available bioinformatics tools for gene list analysis could serve as a standard for education [30, 31], allows to reveal novel features for cancer diseases. The network analysis complements analysis of microarray gene expression and transcriptomics data for glioblastoma research [7, 32]. The network modelling using the described tools proved to be fruitful in other complex disease analysis, such as Parkinson disease, mental disorders, and diabetes [33, 34].
