# Supplementary material for: Glioblastoma gene network reconstruction and ontology analysis by online bioinformatics tools
Source: J Integr Bioinform. 2021 Nov 16;18(4):20210031. doi: 10.1515/jib-2021-0031 (PMC8709738; doi:10.1515/jib-2021-0031)
Supplement: Supplementary file 1 [file jib-18-20210031-s001.docx]

**Supplement**

for “Glioblastoma gene network reconstruction and ontology analysis by online bioinformatics tools” by Gubanova et al.
*Journal of Integrative Bioinformatics*

**List of glioblastoma genes from OMIM.org used in the analysis**

| **Gene Symbol** | **Genomic coordinates (GRCh38)** | **Gene/Locus (aliases)** | **Gene/Locus name** | **MIM number** |
| --- | --- | --- | --- | --- |
| ENO1 | 1:8860999-8878685 | ENO1, PPH, MPB1 | Enolase-1, alpha | 172430 |
| MTOR | 1:11106534-11273496 | MTOR, FRAP1, SKS | Mechanistic target of rapamycin | 601231 |
| MIIP | 1:12019465-12032044 | MIIP, IIP45 | Migration and invasion inhibitory protein | 608772 |
| PLA2G2A | 1:19975430-19980433 | PLA2G2A, PLA2B, PLA2L, MOM1 | Phospholipase A2, group IIA, platelets, synovial fluid | 172411 |
| ASAP3 | 1:23428562-23484630 | ASAP3, UPLC1, CENTB6, DDEFL1, ACAP4 | ARF GTPase-activating protein with SH3 domain, ankyrin repeat, and PH domain 3 | 616594 |
| ADGRB2 | 1:31727104-31764339 | ADGRB2, BAI2 | Adhesion G protein-coupled receptor B2 | 602683 |
| RBBP4 | 1:32651207-32686210 | RBBP4, RBAP48 | Retinoblastoma-binding protein 4 | 602923 |
| HMGB4 | 1:33860474-33864790 | HMGB4 | High mobility group box 4 | 617285 |
| CAP1 | 1:40040239-40072647 | CAP1 | Cyclase-associated actin cytoskeleton regulatory protein 1 | 617801 |
| SLC2A1 | 1:42925352-42958867 | SLC2A1, GLUT1, HTLVR, DYT18, PED, GLUT1DS, EIG12, DYT9, SDCHCN | Solute carrier family 2 (facilitated glucose transporter), member 1 | 138140 |
| PTCH2 | 1:44819844-44843252 | PTCH2 | Patched 2 | 603673 |
| PDE4B | 1:65792509-66374578 | PDE4B, DPDE4 | Phosphodiesterase-4B, cAMP-specific (dunce-like phosphodiesterase E4) | 600127 |
| NRAS | 1:114704468-114716770 | NRAS, ALPS4, NS6, CMNS, NCMS | NRAS protooncogene, GTPase | 164790 |
| APH1A | 1:150265403-150282178 | APH1A | APH1 homolog A, gamma secretase subunit | 607629 |
| S100A16 | 1:153606882-153613136 | S100A16, AAG13 | S100 calcium-binding protein A16 | 617437 |
| ASPM | 1:197084126-197146668 | ASPM, MCPH5 | Abnormal spindle-like, microcephaly-associated | 605481 |
| MIR181B1 | 1:198858872-198858981 | MIR181B1, MIRN181B1 | Micro RNA 181B1 | 612744 |
| MIR181A1 | 1:198859043-198859152 | MIR181A1, MIR213, MIRN181A1 | Micro RNA 181A1 | 612742 |
| LRRN2 | 1:204617169-204685835 | LRRN2, GAC1, LRANK1 | Leucine-rich repeat protein, neuronal, 2 | 605492 |
| KLHDC8A | 1:205336060-205357038 | KLHDC8A | Kelch domain-containing protein 8A | 614503 |
| TGFB2 | 1:218345335-218444618 | TGFB2, LDS4 | Transforming growth factor, beta-2 | 190220 |
| CAPN2 | 1:223701596-223776017 | CAPN2 | Calpain, large polypeptide L2 | 114230 |
| ENAH | 1:225486828-225660045 | ENAH, ENA, MENA, NDPP1 | Enabled, Drosophila, homolog of | 609061 |
| H3-3A | 1:226061830-226072018 | H3F3A, H3F3 | H3 histone, family 3A | 601128 |
| LIN9 | 1:226231148-226310081 | LIN9 | Lin9, C. elegans, homolog of | 609375 |
| COQ8A | 1:226939338-226987543 | ADCK3, COQ8, CABC1, SCAR9, ARCA2, COQ10D4 | AARF domain-containing kinase 3 | 606980 |
| GJC2 | 1:228149929-228159825 | GJC2, GJA12, CX47, PMLDAR, HLD2, SPG44, LMPHM3 | Gap junction protein, gamma-2 (47kD) | 608803 |
| PXDN | 2:1631886-1744851 | PXDN, D2S448E, KIAA0230, PRG2, PXN, COPOA, ASGD7 | Peroxidasin | 605158 |
| ID2 | 2:8682055-8684460 | ID2 | Inhibitor of DNA binding 2, dominant negative | 600386 |
| ADAM17 | 2:9488485-9555829 | ADAM17, TACE, NISBD1 | ADAM metallopeptidase domain 17 | 603639 |
| ATAD2B | 2:23687623-23927122 | ATAD2B, KIAA1240 | ATPase family, AAA domain-containing, member 2B | 615347 |
| EPAS1 | 2:46297406-46386696 | EPAS1, MOP2, HIF2A, ECYT4 | Endothelial PAS domain protein 1 | 603349 |
| MSH2 | 2:47403066-47634500 | MSH2, COCA1, FCC1, HNPCC1, MMRCS2 | mutS homolog 2 | 609309 |
| MSH6 | 2:47783144-47806953 | MSH6, GTBP, HNPCC5, MMRCS3 | mutS homolog 6 | 600678 |
| RTKN | 2:74425834-74441936 | RTKN | Rhotekin | 602288 |
| EVA1A | 2:75492317-75569718 | EVA1A, TMEM166, FAM176A | EVA1 homolog A, regulator of programmed cell death | 618990 |
| BUB1 | 2:110637527-110678062 | BUB1 | BUB1 mitotic checkpoint serine/threonine kinase | 602452 |
| MIR128-1 | 2:135665396-135665477 | MIR128-1, MIRN128-1, MIR128A | Micro RNA 128-1 | 611774 |
| TTC21B | 2:165873361-165953780 | TTC21B, THM1, NPHP12, SRTD4, ATD4 | Tetratricopeptide repeat domain 21B | 612014 |
| MIR10B | 2:176150302-176150411 | MIR10B, MIRN10B | Micro RNA 10B | 611576 |
| NBEAL1 | 2:203013783-203225193 | NBEAL1, ALS2CR17 | Neurobeachin-like 1 | 609816 |
| CTLA4 | 2:203867770-203873964 | CTLA4, IDDM12, CELIAC3, ALPS5 | Cytotoxic T-lymphocyte-associated serine esterase-4 | 123890 |
| IDH1 | 2:208236226-208255070 | IDH1 | Isocitrate dehydrogenase, soluble | 147700 |
| VHL | 3:10141777-10153666 | VHL | von Hippel-Lindau tumor suppressor | 608537 |
| PPARG | 3:12287367-12434343 | PPARG, PPARG1, PPARG2, CIMT1, GLM1 | Peroxisome proliferator activated receptor, gamma | 601487 |
| RAF1 | 3:12583600-12664116 | RAF1, CRAF, NS5, CMD1NN | Oncogene RAF1 | 164760 |
| IQSEC1 | 3:12897042-13283256 | IQSEC1, KIAA0763, IDDSSBA | IQ motif- and Sec7 domain-containing protein 1 | 610166 |
| NR1D2 | 3:23945285-23980617 | NR1D2, RVR, BD73 | Nuclear receptor subfamily 1, group D, member 2 | 602304 |
| MLH1 | 3:36993486-37050845 | MLH1, COCA2, HNPCC2, MMRCS1 | DNA mismatch repair protein MLH1 | 120436 |
| RPSA | 3:39406719-39412541 | RPSA, LAMR1, LAMBR, ICAS | Ribosomal protein SA | 150370 |
| CTNNB1 | 3:41199421-41240444 | CTNNB1, NEDSDV, EVR7 | Catenin (cadherin-associated protein), beta 1, 88kD | 116806 |
| HIGD1A | 3:42782907-42804489 | HIGD1A, HIG1, RCF1A | HIG1 hypoxia-inducivle domain family, member 1A | 618623 |
| RIOX2 | 3:97941817-97972430 | RIOX2, MINA, MINA53, MDIG | Ribosomal oxygenase 2 | 612049 |
| IL12A | 3:159988834-159996018 | IL12A | Interleukin-12A (natural killer cell stimulatory factor-1, cytotoxic lymphocyte maturation factor-1, p35) | 161560 |
| BCHE | 3:165772903-165837422 | BCHE, CHE1, BCHED | Butyrylcholinesterase | 177400 |
| PIK3CA | 3:179148113-179240092 | PIK3CA, CLOVE, MCAP, MCM, MCMTC, CWS5, CLAPO, CCM4 | Phosphatidylinositol 3-kinase, catalytic, alpha polypeptide | 171834 |
| TACC3 | 4:1721496-1745170 | TACC3 | Transforming, acidic, coiled-coil-containing protein 3 | 605303 |
| FGFR3 | 4:1793292-1808871 | FGFR3, ACH | Fibroblast growth factor receptor-3 | 134934 |
| PROM1 | 4:15968225-16084058 | PROM1, PROML1, AC133, RP41, CORD12, CD133, MCDR2, STGD4 | Prominin 1 | 604365 |
| PDGFRA | 4:54229126-54298244 | PDGFRA | Platelet-derived growth factor receptor, alpha polypeptide | 173490 |
| KDR | 4:55078480-55125594 | KDR | Kinase insert domain receptor | 191306 |
| LIN54 | 4:82924602-83012950 | LIN54 | Lin54, C. elegans, homolog of | 613367 |
| OSTC | 4:108650598-108667819 | OSTC | Oligosaccharyltransferase complex, noncatalytic subunit | 619023 |
| TLR2 | 4:153684079-153710642 | TLR2, TIL4 | Toll-like receptor-2 | 603028 |
| KLHL2 | 4:165207560-165323155 | KLHL2, MAYVEN | Kelch-like 2 | 605774 |
| VEGFC | 4:176683537-176792921 | VEGFC, VRP, LMPHM4 | Vascular endothelial growth factor C | 601528 |
| FAT1 | 4:186587788-186726695 | FAT1, FAT | FAT atypical cadherin 1 | 600976 |
| TERT | 5:1253166-1295067 | TERT, TCS1, EST2, DKCA2, DKCB4, PFBMFT1, CMM9 | Telomerase reverse transcriptase | 187270 |
| OSMR | 5:38846011-38945578 | OSMR, OSMRB, PLCA1 | Oncostatin M receptor | 601743 |
| IL31RA | 5:55840333-55922853 | IL31RA, GLMR, GPL, PLCA2 | Interleukin 31 receptor A | 609510 |
| NDUFAF2 | 5:60945204-61153025 | NDUFAF2, NDUFA12L, MMTN, B17.2L, MC1DN10 | NADH-ubiquinone oxidoreductase complex assembly factor 2 | 609653 |
| PIK3R1 | 5:68215736-68301820 | PIK3R1, GRB1, AGM7, SHORT, IMD36 | Phosphatidylinositol 3-kinase, regulatory, 1 | 171833 |
| MIR9-2 | 5:88666852-88666938 | MIR9-2, MIRN9-2 | Micro RNA 9-2 | 611187 |
| APC | 5:112707497-112846238 | APC, GS, FPC, BTPS2, DESMD | APC regulator of WNT signaling pathway | 611731 |
| MCC | 5:113022105-113488452 | MCC | MCC regulator of WNT signaling pathway | 159350 |
| IL12B | 5:159314779-159330486 | IL12B, NKSF2, IMD29 | Interleukin-12B (natural killer cell stimulatory factor-2, cytotoxic lymphocyte maturation factor-2, p40) | 161561 |
| CPEB4 | 5:173888327-173961979 | CPEB4, KIAA1673 | Cytoplasmic polyadenylation element-binding protein 4 | 610607 |
| ATXN1 | 6:16299111-16761489 | ATXN1, ATX1, SCA1 | Ataxin-1 | 601556 |
| CAP2 | 6:17393594-17557779 | CAP2 | Cyclase-associated actin cytoskeleton regulatory protein 2 | 618385 |
| RNF144B | 6:18387328-18468869 | RNF144B | RING finger protein 144B | 618869 |
| CARMIL1 | 6:25279373-25620529 | CARMIL1, LRRC16A, LRRC16 | Capping protein regulator and myosin 1 linker 1 | 609593 |
| H3C2 | 6:26031588-26032098 | HIST1H3B, H3FL | Histone 1, H3fl | 602819 |
| HLA-G | 6:29826473-29831129 | HLA-G | HLA-G histocompatibility antigen, class I | 142871 |
| DAXX | 6:33318557-33322958 | DAXX | Death-associated protein 6 | 603186 |
| DAAM2 | 6:39792365-39904869 | DAAM2, KIAA0381, NPHS24 | Dishevelled-associated activator of morphogenesis 2 | 606627 |
| VEGFA | 6:43770208-43786486 | VEGF, MVCD1 | Vascular endothelial growth factor | 192240 |
| TMEM14A | 6:52671098-52686587 | TMEM14A | Transmembrane protein 14A | 616870 |
| HMGCLL1 | 6:55434372-55607572 | HMGCLL1, ERCHL | 3-hydroxy-3-methylglutaryl-CoA lyase-like protein 1 | 619050 |
| PHF3 | 6:63635801-63726010 | PHF3, KIAA0244 | PHD finger protein 3 | 607789 |
| ADGRB3 | 6:68635281-69389505 | ADGRB3, BAI3 | Adhesion G protein-coupled receptor B3 | 602684 |
| ROS1 | 6:117287352-117426064 | ROS1, MCF3 | Avian UR2 sarcoma virus oncogene (v-ros) homolog 1 | 165020 |
| GOPC | 6:117560268-117602519 | GOPC, PIST, FIG, CAL | Golgi-associated PDZ and coiled-coil domains-containing protein | 606845 |
| FABP7 | 6:122749200-122784073 | FABP7, FABPB, BLBP | Fatty acid-binding protein 7 | 602965 |
| RNF146 | 6:127263414-127289892 | RNF146 | Ring finger protein 146 | 612137 |
| SYNJ2 | 6:157981855-158099175 | SYNJ2, KIAA0348 | Synaptojanin 2 | 609410 |
| PRKN | 6:161347416-162727801 | PRKN, PARK2, PDJ | Parkin | 602544 |
| MIR339 | 7:1022932-1023025 | MIR339 | Micro RNA 339 | 615977 |
| MAD1L1 | 7:1815794-2232944 | MAD1L1, TXBP181 | Mitotic arrest-deficient 1, yeast, homolog-like 1 | 602686 |
| PMS2 | 7:5970924-6009105 | PMS2, PMSL2, HNPCC4, MMRCS4 | PMS1 homolog 2, mismatch repair system component | 600259 |
| RAC1 | 7:6374526-6403966 | RAC1, MRD48 | Rac family small GTPase 1 | 602048 |
| TMEM106B | 7:12211293-12243366 | TMEM106B, HLD16 | Transmembrane protein 106B | 613413 |
| HNRNPA2B1 | 7:26189919-26200774 | HNRPA2B1, IBMPFD2 | Heterogeneous nuclear ribonucleoprotein A2/B1 | 600124 |
| CDK13 | 7:39949663-40099579 | CDK13, CDC2L5, CHED, CHDFIDD | Cyclin-dependent kinase 13 | 603309 |
| GLI3 | 7:41960948-42237208 | GLI3, PAPA, PAPB | GLI-Kruppel family member GLI3 (oncogene GLI3) | 165240 |
| EGFR | 7:55019016-55211627 | EGFR, NISBD2 | Epidermal growth factor receptor | 131550 |
| VOPP1 | 7:55434405-55572501 | VOPP1, ECOP, GASP | Vesicular, overexpressed in cancer, prosurvival protein 1 | 611915 |
| SEPTIN14 | 7:55793539-55862751 | SEPT14 | Septin 14 | 612140 |
| NIPSNAP2 | 7:55964584-56000178 | NIPSNAP2, GBAS | Nipsnap homolog 2 | 603004 |
| PTPN12 | 7:77537189-77640068 | PTPN12, PTPG1 | Protein tyrosine phosphatase, nonreceptor-type, 12 | 600079 |
| HGF | 7:81699005-81770437 | HGF, DFNB39 | Hepatic growth factor | 142409 |
| CDK6 | 7:92604920-92836572 | CDK6, PLSTIRE, MCPH12 | Cyclin-dependent kinase 6 | 603368 |
| MIR25 | 7:100093559-100093642 | MIR25, MIRN25 | Micro RNA 25 | 612150 |
| EPHB4 | 7:100802564-100827522 | EPHB4, HTK, MYK1, HFASD, CMAVM2, LMPHM7 | Ephrin receptor EphB4 (hepatoma transmembrane kinase) | 600011 |
| PTPRZ1 | 7:121873160-122062035 | PTPRZ1, PTP18 | Protein-tyrosine phosphatase, receptor-type, zeta-1, polypeptide | 176891 |
| LRRC4 | 7:128027070-128032106 | LRRC4 | Leucine-rich repeat-containing protein 4 | 610486 |
| BRAF | 7:140713327-140924928 | BRAF, NS7 | B-Raf proto-oncogene, serine/threonine kinase | 164757 |
| ANGPT2 | 8:6499631-6563244 | ANGPT2, ANG2, LMPHM10 | Angiopoietin 2 | 601922 |
| CTSB | 8:11842523-11868086 | CTSB, CPSB, RECEUP | Cathepsin B (regulatory element, cis-acting, enhancer upstream of CTSB, included) | 116810 |
| DLC1 | 8:13083360-13604619 | DLC1 | DLC1 Rho GTPase activating protein | 604258 |
| PDGFRL | 8:17576432-17643143 | PDGFRL, PDGRL, PRLTS | Platelet-derived growth factor receptor-like | 604584 |
| PURG | 8:30995801-31033355 | PURG, PURGA, PURGB | Purine-rich element-binding protein G | 618041 |
| DUSP26 | 8:33591329-33600022 | DUSP26, MKP8, LDP4, NEAP | Dual-specificity phosphatase 26 | 618368 |
| FGFR1 | 8:38411142-38468634 | FGFR1, FLT2, OGD, KAL2, HH2, HRTFDS, ECCL | Fibroblast growth factor receptor-1 (fms-related tyrosine kinase-2) | 136350 |
| TACC1 | 8:38728204-38853027 | TACC1 | Transforming, acidic, coiled-coil-containing protein-1 | 605301 |
| ADAM9 | 8:38996766-39105260 | ADAM9, MDC9, MCMP, CORD9 | ADAM metallopeptidase domain 9 | 602713 |
| PDE7A | 8:65714333-65842063 | PDE7A, HCP1 | Phosphodiesterase-7A | 171885 |
| PI15 | 8:74824533-74855028 | PI15, P25TI | Protease inhibitor 15 | 607076 |
| RAD54B | 8:94371959-94475114 | RAD54B | RAD54 homolog B | 604289 |
| ADGRB1 | 8:142449648-142545008 | ADGRB1, BAI1, GDAIF | Adhesion G protein-coupled receptor B1 | 602682 |
| MAF1 | 8:144104460-144107610 | MAF1 | MAF1 homolog, negative regulator of RNA polymerase III | 610210 |
| WASHC1 | 9:14474-30486 | WASHC1, WASH1 | WASH complex, subunit 1 | 613632 |
| CD274 | 9:5450541-5470553 | CD274, PDCD1LG1, B7H1 | CD274 molecule | 605402 |
| GLDC | 9:6532466-6645728 | GLDC, HYGN1, GCSP, GCE, NKH | Glycine dehydrogenase (decarboxylating; glycine decarboxylase, glycine cleavage system protein P) | 238300 |
| FOCAD | 9:20658308-20995952 | FOCAD, KIAA1797 | Focadhesin | 614606 |
| CDKN2A | 9:21967751-21995323 | CDKN2A, MTS1, P16, MLM, CMM2 | Cyclin-dependent kinase inhibitor 2A (p16, inhibits CDK4) | 600160 |
| CNTNAP3 | 9:39064709-39288166 | CNTNAP3, KIAA1714 | Contactin-associated protein-like 3 | 610517 |
| NTRK2 | 9:84668457-85027069 | NTRK2, TRKB, OBHD, DEE58 | Neurotrophic tyrosine kinase, receptor, type 2 | 600456 |
| MIR181A2 | 9:124692441-124692550 | MIR181A2, MIRN181A2 | Micro RNA 181A2 | 612743 |
| MIR181B2 | 9:124693709-124693797 | MIR181B2, MIRN181B2 | Micro RNA 181B2 | 612745 |
| UBAC1 | 9:135932968-135961372 | UBAC1, GBDR1 | Ubiquitin-associated domain-containing protein 1 | 608129 |
| MIR126 | 9:136670601-136670685 | MIR126, MIRN126 | Micro RNA 126 | 611767 |
| MLLT10 | 10:21524615-21743629 | AF10 | ALL1 fused gene from chromosome 10 | 602409 |
| PTEN | 10:87863624-87971929 | PTEN, MMAC1, GLM2, CWS1 | Phosphatase and tensin homolog (mutated in multiple advanced cancers 1) | 601728 |
| LGI1 | 10:93757886-93798158 | LGI1, EPT, ETL1, ADLTE, ADPEAF | Leucine-rich gene, glioma-inactivated, 1 | 604619 |
| OGA | 10:101784444-101818708 | OGA, MGEA5, NCOAT | O-GlcNAcase | 604039 |
| SUFU | 10:102502800-102633534 | SUFU, SUFUXL, SUFUH, JBTS32 | Suppressor of fused | 607035 |
| TRIM8 | 10:102644478-102658318 | TRIM8, RNF27, GERP, FSGSNEDS | Tripartite motif-containing 8 | 606125 |
| MXI1 | 10:110207604-110287364 | MXI1 | MAX-interacting protein 1 | 600020 |
| WDR11 | 10:120851312-120909524 | WDR11, DR11, KIAA1351, BRWD2, HH14 | WD repeat-containing protein 11 | 606417 |
| DMBT1 | 10:122560676-122643735 | DMBT1 | Deleted in malignant brain tumors 1 | 601969 |
| MGMT | 10:129467240-129770982 | MGMT | Methylguanine-DNA methyltransferase | 156569 |
| BRSK2 | 11:1389933-1462688 | BRSK2, SAD1, PEN11B | BR serine/threonine kinase 2 | 609236 |
| PHLDA2 | 11:2928272-2929419 | PHLDA2, TSSC3, IPL, BRW1C | Pleckstrin homology-like domain, family A, member 2 | 602131 |
| DKK3 | 11:11963035-12009826 | DKK3, RIG | Dickkopf WNT signaling pathway inhibitor 3 | 605416 |
| RASSF10 | 11:13009315-13012118 | RASSF10 | Ras association domain family, member 10 | 614713 |
| PAX6 | 11:31789025-31817960 | PAX6, AN2, MGDA, FVH1, ASGD5 | Paired box homeotic gene-6 | 607108 |
| PTPRJ | 11:47980503-48170838 | PTPRJ, DEP1 | Protein tyrosine phosphatase, receptor type, J polypeptide | 600925 |
| MIR130A | 11:57641197-57641285 | MIR130A, MIRN130A | Micro RNA 130A | 610175 |
| FAM111A | 11:59142855-59155038 | FAM111A, KIAA1895, KCS2, GCLEB | Family with sequence similarity 111, member A | 615292 |
| TMEM132A | 11:60924459-60937158 | TMEM132A, KIAA1583 | Transmembrane protein 132A | 617363 |
| CHKA | 11:68052858-68121388 | CHKA, CHK | Choline kinase, alpha | 118491 |
| CCND1 | 11:69641155-69654473 | CCND1, PRAD1, BCL1 | Cyclin D1 | 168461 |
| MIR326 | 11:75335091-75335185 | MIR326, MIRN326 | Micro RNA 326 | 613755 |
| BACE1 | 11:117285697-117316255 | BACE1, BACE | Beta-site amyloid beta A4 precursor protein-cleaving enzyme (secretase, beta; memapsin 2) | 604252 |
| MIR125B1 | 11:122099756-122099843 | MIR125B1, MIRN125B1 | Micro RNA 125B-1 | 610104 |
| MIRLET7A2 | 11:122146521-122146592 | MIRLET7A2, LET7A2, MIRNLET7A2 | Micro RNA let7a2 | 612142 |
| MIR100 | 11:122152228-122152307 | MIR100, MIRN100 | Micro RNA 100 | 613186 |
| IQSEC3 | 12:66766-178454 | IQSEC3, KIAA1110 | IQ motif- and SEC7 domain-containing protein 3 | 612118 |
| KDM5A | 12:280056-389319 | KDM5A, JARID1A, RBP2, RBBP2 | Lysine demethylase 5A | 180202 |
| ING4 | 12:6650300-6663142 | ING4 | Inhibitor of growth-4 | 608524 |
| ENO2 | 12:6914579-6923696 | ENO2 | Enolase-2, gamma, neuronal | 131360 |
| KRAS | 12:25205245-25250928 | KRAS, KRAS2, RASK2, NS, CFC2, RALD, OES | KRAS protooncogene, GTPase | 190070 |
| SHMT2 | 12:57229684-57234934 | SHMT2, GLYA, NEDCASB | Serine hydroxymethyltransferase | 138450 |
| YEATS4 | 12:69359704-69427076 | YEATS4, GAS41 | YEATS domain-containing protein 4 | 602116 |
| GLIPR1 | 12:75480753-75503862 | GLIPR1, GLIPR, RTVP1 | Glioma pathogenesis-related protein 1 | 602692 |
| MTERF2 | 12:106972855-106987165 | MTERF2, MTERFD3 | Transcription termination factor 2, mitochondrial | 616929 |
| MSI1 | 12:120339661-120369173 | MSI1 | Musashi RNA binding protein 1 | 603328 |
| BRCA2 | 13:32315507-32400267 | BRCA2, FANCD1, BROVCA2, GLM3, PNCA2 | BRCA2 DNA repair-associated protein | 600185 |
| RB1 | 13:48303750-48481889 | RB1 | Retinoblastoma-1 | 614041 |
| ARHGEF7 | 13:111114618-111305733 | ARHGEF7, PIXB, COOL1 | Rho guanine nucleotide exchange factor 7 (PAK-interacting exchange factor, beta) | 605477 |
| NFKBIA | 14:35401512-35404748 | NFKBIA, IKBA, EDAID2 | Nuclear factor kappa-B inhibitor, alpha | 164008 |
| BMP4 | 14:53949735-53956890 | BMP4, BMP2B1, BMP2B, MCOPS6, OFC11 | Bone morphogenetic protein-4 | 112262 |
| ARID4A | 14:58298384-58373875 | ARID4A, RBP1, RBBP1 | AT-rich interactive domain-containing protein 4A | 180201 |
| VTI1B | 14:67647084-67674631 | VTI1B, VTI1, VTI1L | Vesicle transport through interaction with T-snares 1B | 603207 |
| MLH3 | 14:75013763-75051478 | MLH3, HNPCC7 | DNA mismatch repair gene MLH3 | 604395 |
| FOXN3 | 14:89156176-89619164 | FOXN3, CHES1 | Forkhead box N3 | 602628 |
| AKT1 | 14:104769348-104795747 | AKT1, CWS6 | AKT serine/threonine kinase 1 | 164730 |
| BUB1B | 15:40161068-40221122 | BUB1B, BUBR1, MVA1 | BUB1 mitotic checkpoint serine/threonine kinase B | 602860 |
| MYEF2 | 15:48134631-48178516 | MYEF2, KIAA1341 | Myelin expression factor 2 | 619395 |
| SECISBP2L | 15:48988637-49046445 | SECISBP2L, SBP2L, KIAA0256 | Selenocysteine insertion sequence-binding protein 2-like | 615756 |
| ADAM10 | 15:58588808-58749706 | ADAM10, MADM, RAK, AD18 | ADAM metallopeptidase domain 10 | 602192 |
| APH1B | 15:63277549-63309125 | APH1B | Aph-1 homolog B, gamma-secretase subunit | 607630 |
| PKM | 15:72199028-72231388 | PKM, PKM2, PK3, THBP1 | Pyruvate kinase, muscle | 179050 |
| NEO1 | 15:73051714-73305205 | NEO1, NGN | Neogenin, chicken, homolog of, 1 | 601907 |
| IDH2 | 15:90083044-90102467 | IDH2, IDPM, D2HGA2 | Isocitrate dehydrogenase, mitochondrial | 147650 |
| CHSY1 | 15:101175726-101252047 | CHSY1, KIAA0990, TPBS | Carbohydrate synthase 1 | 608183 |
| PKD1 | 16:2088707-2135897 | PKD1 | Polycystin-1 | 601313 |
| MMP25 | 16:3,045,962 | MMP25, MMP20A | Matrix metalloproteinase 25 | 608482 |
| FOPNL | 16:15,865,718 | FOPNL, FOR20, C16orf63 | FGFR1OP N-terminal domain-like protein | 617149 |
| CCP110 | 16:19,523,878 | CCP110, CP110, KIAA0419 | Centrosomal coiled-coil protein, 110kD | 609544 |
| PLK1 | 16:23,678,888 | PLK1, STPK13 | Polo-like kinase 1 | 602098 |
| NUDT21 | 16:56,429,132 | NUDT21, CPSF5, CFIM25 | Nudix hydrolase 21 | 604978 |
| NDRG4 | 16:58,463,703 | NDRG4, SMAP8, KIAA1180 | NMYC downstream-regulated gene 4 | 614463 |
| TAX1BP3 | 17:3,662,894 | TAX1BP3, TIP1 | TAX1-binding protein 3 | 616484 |
| ANKFY1 | 17:4,163,819 | ANKFY1, ANKHZN, KIAA1255 | Ankyrin repeats- and FYVE domain-containing protein 1 | 607927 |
| TP53 | 17:7,668,420 | TP53, P53, LFS1 , BCC7, BMFS5 | Tumor protein p53 | 191170 |
| FLCN | 17:17,206,945 | FLCN, BHD | Folliculin | 607273 |
| SDF2 | 17:28,648,345 | SDF2 | Stromal cell-derived factor-2 | 602934 |
| NF1 | 17:31,094,926 | NF1, VRNF, WSS, NFNS | Neurofibromin (neurofibromatosis, type I) | 613113 |
| ERBB2 | 17:39,688,093 | ERBB2, NGL, NEU, HER2, VSCN2 | Avian erythroblastic leukemia viral (v-erb-b2) oncogene homolog 2 (neuro/glioblastoma derived oncogene homolog) | 164870 |
| CSF3 | 17:40,015,439 | CSF3, GCSF | Colony-stimulating factor-3 (granulocyte) | 138970 |
| NR1D1 | 17:40,092,792 | NR1D1, THRAL, EAR1 | Nuclear receptor 1, subfamily D, member 1 (thyroid hormone receptor, alpha-1-like) | 602408 |
| STAT3 | 17:42,313,323 | STAT3, APRF, HIES, ADMIO1 | Signal transducer and activator of transcription-3 (acute-phase response factor) | 102582 |
| MIR21 | 17:59,841,265 | MIR21, MIRN21 | Micro RNA 21 | 611020 |
| AXIN2 | 17:65,528,562 | AXIN2, ODCRCS | Axis inhibitor 2 (conductin, mouse, homolog of) | 604025 |
| PCYT2 | 17:81,900,957 | PCYT2, ET, SPG82 | Phosphate cytidylyltransferase 2, ethanolamine | 602679 |
| DCC | 18:52,340,171 | DCC, MRMV1, HGPPS2 | Deleted in colorectal carcinoma | 120470 |
| PHLPP1 | 18:62,715,444 | PHLPP1, SCOP, KIAA0606 | PH domain and leucine-rich repeat protein phosphatase | 609396 |
| MBP | 18:76,978,832 | MBP | Myelin basic protein | 159430 |
| STK11 | 19:1,205,777 | STK11, PJS, LKB1 | Serine/threonine protein kinase-11 | 602216 |
| SEMA6B | 19:4,542,587 | SEMA6B, EPM11 | Semaphorin 6B | 608873 |
| ICAM1 | 19:10,271,119 | ICAM1 | Intercellular adhesion molecule-1 | 147840 |
| RAD3D | 19:11,322,067 | RAD3D, GOV | Ras family, member RAB3D | 604350 |
| MIR181C | 19:13,874,698 | MIR181C, MIRN181C | Micro RNA 181C | 612746 |
| CERS1 | 19:18,868,544 | CERS1, LASS1, UOG1, EPM8 | Ceramide synthase 1 | 606919 |
| KMT2B | 19:35,717,817 | KMT2B, MLL4, KIAA0304, DYT28 | Lysine-specific methyltransferase 2B | 606834 |
| HNRNPL | 19:38,836,369 | HNRNPL, HNRPL | Heterogeneous nuclear riboprotein 1 | 603083 |
| DYRK1B | 19:39,825,349 | DYRK1B, MIRK, AOMS3 | Dual-specificity tyrosine phosphorylation-regulated kinase 1B | 604556 |
| DEDD2 | 19:42,198,597 | DEDD2, FLAME3 | Death effector domain-containing protein 2 | 617078 |
| BAX | 19:48,954,824 | BAX | BCL2-associated X protein | 600040 |
| BCL2L12 | 19:49,664,833 | BCL2L12 | BCL2-like 12 | 610837 |
| EMC10 | 19:50,476,482 | EMC10, C19orf63, HSS1, HSM1, NEDDFAS | ER membrane protein complex subunit 10 | 614545 |
| ZNF320 | 19:52,859,490 | ZNF320 | Zinc finger protein 320 | 606427 |
| RBBP9 | 20:18,486,539 | RBBP9, BOG | Retinoma-binding protein 9 | 602908 |
| CST9 | 20:23,602,409 | CST9, CLM | Cystatin 9 | 616543 |
| PHF20 | 20:35,772,002 | PHF20, GLEA2, HCA58 | PHD finger protein 20 | 610335 |
| SRC | 20:37,344,689 | SRC, ASV, SRC1, THC6 | Protooncogene SRC, Rous sarcoma | 190090 |
| MYBL2 | 20:43,667,113 | MYBL2, BMYB | v-myb avian myeloblastosis viral oncogene homolog-like 2 | 601415 |
| TP53TG5 | 20:45,372,556 | TP53TG5 | TP53 target gene 5 | 617316 |
| AURKA | 20:56,369,389 | AURKA, STK15, AURORA2, BTAK, ARK1, STK6, AIK | Aurora kinase A | 603072 |
| MIR99A | 21:16,539,088 | MIR99A | Micro RNA 99A | 614509 |
| MIRLET7C | 21:16,539,827 | MIRLET7C, MIRNLET7C, LET7C | Micro RNA Let7c | 612144 |
| MIR125B2 | 21:16,590,236 | MIR125B2, MIRN125B2 | Micro RNA 125B-2 | 610105 |
| GABPA | 21:25,734,971 | GABPA, E4TF1A | GA-binding protein transcription factor, alpha subunit, 60kD | 600609 |
| OLIG2 | 21:33,025,934 | OLIG2, PRKCBP2 | Oligodendrocyte lineage transcription factor 2 | 606386 |
| RCAN1 | 21:34,516,441 | RCAN1, DSCR1, MCIP1, CSP1 | Regulator of calcineurin 1 | 602917 |
| DYRK1A | 21:37,365,572 | DYRK1A, MNBH, MNB, MRD7 | Dual specificity tyrosine-(Y)-phosphorylation regulated kinase-1A (\'minibrain\', Drosophila, homolog of) | 600855 |
| CHEK2 | 22:28,687,742 | CHEK2, RAD53, CHK2, CDS1, LFS2 | Checkpoint kinase 2 | 604373 |
| PES1 | 22:30,576,624 | PES1, PES | Pescadillo, zebrafish, homolog of, 1 | 605819 |
| PDGFB | 22:39,223,358 | PDGFB, SIS, IBGC5 | Platelet-derived growth factor, beta polypeptide (oncogene SIS) | 190040 |
| EP300 | 22:41,092,591 | EP300, RSTS2, MKHK2 | E1A-binding protein, 300kD | 602700 |
| PLXNB2 | 22:50,274,978 | PLXNB2, MM1 | Plexin B2 | 604293 |
| MIR221 | X:45,746,156 | MIR221, MIRN221 | Micro RNA 221 | 300568 |
| MIR222 | X:45,747,014 | MIR222, MIRN222 | Micro RNA 222 | 300569 |
| PRAF2 | X:49,071,160 | PRAF2, JM4 | PRA1 domain family, member 2 | 300840 |
| MAGED4 | X:52,184,887 | MAGED4, MAGEE1, KIAA1859 | Melanoma antigen, family D, 4 | 300702 |
| SLC7A3 | X:70,925,578 | SLC7A3, CAT3 | Solute carrier family 7 (cationic amino acid transporter, y+ system), member 3 | 300443 |
| NLGN3 | X:71,144,388 | NLGN3, ASPGX1, AUTSX1 | Neuroligin 3 | 300336 |
| ATRX | X:77,504,879 | ATRX, XH2, XNP, SHS, SFM1, MRXHF1 | ATRX chromatin remodeler | 300032 |
| FAM46D | X:80,335,438 | FAM46D | Family with sequence similarity 46, member D | 300976 |
| STAG2 | X:123,960,559 | STAG2, SA2, MKMS, HPE13 | Stromal antigen 2 | 300826 |
| GPC3 | X:133,535,744 | GPC3, SDYS, SGBS1 | Glypican 3 | 300037 |
| SPANXB1 | X:141,002,593 | SPANXB1, SPANXB | SPANX family, member B1 | 300669 |
| PDZD4 | X:153,802,165 | PDZD4, PDZK4, PDZRN4L, KIAA1444, LU1 | PDZ domain-containing 4 | 300634 |
